# The Role of Meat Lipids in Nutrition and Health: Balancing Benefits and Risks

**DOI:** 10.3390/nu17020350

**Published:** 2025-01-19

**Authors:** José A. M. Prates

**Affiliations:** 1 CIISA—Centro de Investigação Interdisciplinar em Sanidade Animal, Faculdade de Medicina Veterinária, Universidade de Lisboa, Av. da Universidade Técnica, 1300-477 Lisboa, Portugal; japrates@fmv.ulisboa.pt or japrates@edu.ulisboa.pt; 2 Associate Laboratory for Animal and Veterinary Sciences (AL4AnimalS), Av. da Universidade Técnica, 1300-477 Lisboa, Portugal

**Keywords:** meat lipids, nutritional value, fatty acids, cholesterol, lipid oxidation, human health

## Abstract

Meat lipids are determinants of the nutritional, sensory and physiological qualities of meat, encompassing triglycerides, phospholipids, cholesterol and bioactive compounds. These lipids provide essential fatty acids, including omega-3 and omega-6 polyunsaturated fatty acids, critical for metabolic regulation, inflammation control and cognitive health. However, the dual role of meat lipids as essential nutrients and potential contributors to health risks, such as cardiovascular disease and oxidative stress, necessitates a nuanced understanding. This review evaluates meat lipids’ biochemical diversity, nutritional contributions and health implications, balancing their benefits and risks. It examines factors influencing lipid composition, including species, diet and processing methods, emphasising strategies such as omega-3 enrichment and antioxidant supplementation to enhance lipid quality. Advances in functional meat products, such as hybrid formulations combining plant and animal lipids, are highlighted for their potential to improve health outcomes. Emerging technologies in lipidomics provide deeper insights into lipid oxidation pathways and nutritional profiling, aiding in the development of safer, higher-quality meat products. By synthesising recent evidence, this review offers insights into dietary guidelines, optimises consumer choices and informs sustainable meat production practices aligned with public health and environmental goals.

## 1. Introduction

Meat has been a cornerstone of human diets for millennia, valued for its high-quality proteins, vitamins and essential minerals that are challenging to obtain from other sources [1]. Among its components, lipids play a pivotal role in determining meat’s nutritional, sensory and physiological properties. The lipid fraction of meat, which can comprise 1–10% of its weight depending on the species and cut, consists of triglycerides, phospholipids, cholesterol and various bioactive compounds [2]. These lipids significantly influence human health by providing essential nutrients and mediating key metabolic and inflammatory pathways [3].

Lipids in meat serve as a concentrated energy source, delivering 9 kcal per gram, more than twice the energy density of carbohydrates or proteins. They supply essential fatty acids that are critical for human health but cannot be synthesised endogenously. These include omega-3 (*n*-3) and omega-6 (*n*-6) polyunsaturated fatty acids (PUFAs), such as eicosapentaenoic acid (EPA) and docosahexaenoic acid (DHA), which play central roles in cardiovascular health, neurological development and immune regulation [4]. For instance, a daily intake of 250 mg of EPA and DHA is associated with a reduced risk of coronary heart disease [5].

The fatty acid composition of meat varies widely depending on species, diet and processing methods. Grass-fed beef, for example, contains higher levels of omega-3 PUFAs and a more favourable omega-6 to omega-3 (*n*-6/*n*-3) ratio than grain-fed beef. Grass-fed beef can provide up to 1.5 g of omega-3 fatty acids per 100 g of muscle, whereas grain-fed beef averages less than 0.5 g [6]. This ratio is crucial for maintaining homeostasis in inflammatory and metabolic processes, with recommendations suggesting an omega-6 to omega-3 ratio below 4:1 for optimal health outcomes [7].

In addition to PUFAs, meat lipids are a source of bioactive compounds such as conjugated linoleic acid (CLA) and phospholipids. CLA, naturally found in ruminant meat, is associated with lipid-lowering effects, improved body composition and anti-inflammatory properties [8]. A standard serving of beef (100 g) can provide 0.3–0.8 g of CLA, depending on the animal’s diet.

The health implications of meat lipids are complex, involving both potential benefits and risks. While essential fatty acids and bioactive lipids contribute positively to health, excessive intake of certain lipids, such as saturated fatty acids (SFAs) and cholesterol, has historically been linked to adverse outcomes like cardiovascular diseases (CVD). On average, red meat contains 30–40% SFAs, 40–50% monounsaturated fatty acids (MUFAs), and 5–10% PUFAs, although these proportions vary by type and cut [9]. Recent meta-analyses have questioned the strength of the association between dietary SFAs and cardiovascular risk, suggesting that overall diet quality may play a more significant role [10,11,12].

Conversely, lipid oxidation, a process accelerated by cooking and processing, can generate compounds with pro-inflammatory and cytotoxic properties. For example, malondialdehyde and other lipid peroxides formed during meat processing have been linked to oxidative stress, potentially increasing the risk of chronic diseases if consumed in excess [4]. This highlights the importance of preparation methods in modulating the health effects of meat lipids.

This review aims to update and enhance understanding of the role of meat lipids in human health by integrating findings from recent literature, prioritizing studies published within the last 5 years. It focuses on the biochemical composition of meat lipids, their nutritional contributions and their health implications. Meat lipids are examined for their essential roles in energy provision and bioactive functions, as well as potential risks associated with excess consumption or unfavourable fatty acid ratios. The review explores factors influencing meat lipid composition, including animal diet, species and processing methods, with particular attention to strategies such as omega-3 enrichment and antioxidant supplementation to improve lipid quality. Additionally, it addresses the benefits and risks of meat lipid consumption, tackling ongoing controversies surrounding saturated fats, dietary cholesterol and lipid oxidation products. By consolidating recent findings, this review seeks to provide updated, evidence-based insights to inform dietary guidelines, optimize consumer choices and guide future research.

## 2. Biochemical Composition of Meat Lipids

Lipids in meat play a crucial role in determining its nutritional value, sensory properties and physiological effects on human health. Distributed throughout muscle and adipose tissues, these lipids consist of triglycerides, phospholipids, cholesterol and bioactive compounds. Their composition is influenced by factors such as animal species, diet, muscle location and processing methods. In skeletal muscle, lipids are essential for energy storage, cellular integrity and metabolic activity. Depending on the species and muscle type, the lipid content of muscle tissue typically ranges from 1% to 10%, with higher levels found in marbled cuts contributing to flavour and tenderness [13]. Adipose tissue, by contrast, serves as the primary energy depot, containing up to 85% lipids in the form of triglycerides [3].

Meat lipids can be classified into three main categories: triglycerides, phospholipids and cholesterol. Triglycerides, which account for 90–95% of the lipid content in adipose tissue, are neutral lipids composed of three fatty acids esterified to a glycerol backbone. These lipids act as the primary energy reserve and vary significantly based on the animal’s diet, age and breed [14]. Phospholipids, structural lipids primarily located in muscle cell membranes, constitute approximately 1–5% of total lipids and are particularly rich in PUFAs. These lipids play essential roles in cellular fluidity, signal transduction and mitochondrial function [15]. Cholesterol, although present in smaller amounts (approximately 0.3–0.5% of total lipids), is critical for cell membrane stability and serves as a precursor for steroid hormones, bile acids and vitamin D synthesis [8].

The fatty acid profile of meat lipids is a key determinant of their nutritional and health implications. Meat typically contains approximately 40–50% SFAs, 40–45% MUFAs and 5–10% PUFAs [16]. Among SFAs, palmitic acid (16:0) and stearic acid (18:0) are the most abundant, with palmitic acid linked to elevated cholesterol levels, while stearic acid is considered neutral in its cardiovascular effects. MUFAs, primarily represented by oleic acid (18:1), are associated with improved lipid profiles and are considered beneficial for heart health [17]. PUFAs include omega-6 fatty acids, such as linoleic acid (18:2*n*-6), and omega-3 fatty acids, including alpha-linolenic acid (ALA; 18:3*n*-3), EPA (20:5*n*-3) and DHA (22:6*n*-3). The *n*-6/*n*-3 PUFA ratio is particularly important for maintaining inflammatory and metabolic balance, with a recommended ratio below 4:1 for optimal health [18].

Beyond basic lipid classes, meat contains bioactive lipids contributing additional health benefits. CLA, a naturally occurring PUFA in ruminant meat, has anti-inflammatory effects, facilitates lipid metabolism and exerts potential anti-carcinogenic properties. CLA content in meat ranges from 0.3 to 0.8 g per 100 g, with higher levels observed in grass-fed animals due to differences in rumen microbiota activity [8]. Phospholipids, rich in PUFAs, support cell membrane integrity and cognitive function, while branched-chain fatty acids, though present in smaller quantities, have demonstrated immunomodulatory properties and potential roles in metabolic regulation [19]. Emerging research also highlights the potential roles of lipid-derived compounds, such as lysophospholipids and sphingolipids, in antioxidant defence and signalling pathways, emphasizing the complexity and functional diversity of meat lipids [20,21].

## 3. Factors Influencing Meat Lipid Composition

The lipid composition of meat is a dynamic attribute influenced by a combination of animal-related factors, environmental conditions and post-slaughtering processing methods. Understanding these factors is critical for optimizing the nutritional value and health implications of meat products.

Different animal species and breeds have distinct lipid profiles, reflecting variations in metabolism and fat deposition patterns. Ruminants, such as cattle and sheep, produce higher levels of SFAs and CLA due to microbial hydrogenation in the rumen. For example, lamb tends to have a higher CLA concentration than pork or chicken, making it a richer source of bioactive lipids. In contrast, monogastric animals like pigs and poultry reflect the fatty acid composition of their diets more directly, which can be leveraged to enhance their PUFA content (Table 1) [5].

In poultry, the lipid content and fatty acid composition vary significantly between breeds. Slower-growing, heritage breeds often exhibit a more favourable PUFA profile than fast-growing, commercial breeds. For example, free-range chickens tend to have a higher omega-3 content due to access to forage and insects, compared to intensively farmed birds fed grain-based diets [9].

Selective breeding practices within the same species also influence lipid composition. Breeds selected for higher intramuscular fat, or marbling, typically produce meat with greater concentrations of MUFAs, such as oleic acid, which contributes to improved flavour and tenderness. For example, Wagyu beef contains a higher proportion of MUFAs than standard beef breeds, enhancing both sensory quality and potential health benefits [22].

The diet of an animal is one of the most significant factors affecting the lipid profile of its meat. Animals raised on grass-based diets typically exhibit higher levels of omega-3 PUFAs, including ALA, EPA and DHA, compared to animals fed grain-based diets [23]. Additionally, grass-fed animals often have a more favourable *n*-6/*n*-3 ratio.

Supplementing animal diets with specific feed additives can further enrich meat with omega-3 fatty acids. For instance, dietary inclusion of flaxseed, fish oil or algae significantly boosts EPA and DHA concentrations in ruminants. Algae supplementation is particularly effective, as it provides a direct source of DHA, which is challenging to achieve with plant-based feed alone. Studies have shown that algae-enriched diets can double the DHA content in lamb muscle compared to standard diets [3].

Antioxidant supplementation is another dietary strategy to improve the quality of meat lipids. Feeding animals antioxidants like vitamin E or selenium can reduce lipid oxidation, preserving the integrity of PUFAs during storage and cooking. This not only enhances the shelf life of meat but also maintains its nutritional value, as lipid oxidation is a primary cause of PUFA degradation [13].

Post-slaughtering processing and culinary practices significantly affect the final lipid composition of meat. High-temperature cooking methods, such as grilling, frying or roasting, can lead to lipid oxidation, particularly in PUFAs. Oxidized lipids generate undesirable compounds like malondialdehyde, which degrade the nutritional quality of the meat and may contribute to inflammation and oxidative stress when consumed in excess [13].

Curing and smoking processes, commonly used in preserved meats, alter lipid profiles by introducing nitrites and other reactive compounds. While these methods enhance flavour and shelf life, they can increase the formation of nitrosamines, which have been linked to potential health risks [24]. In addition, marinades containing unsaturated oils, herbs and spices can improve the lipid profile of meat by increasing its MUFA and PUFA content and introducing natural antioxidants that mitigate oxidative damage during cooking [4].

Emerging technologies, such as sous vide cooking, offer promising alternatives to traditional methods. By cooking meat at lower temperatures for extended periods, sous vide minimizes lipid oxidation while preserving the nutritional quality and sensory properties of meat [25]. This technique is particularly effective for lean cuts with higher PUFA content, which are more susceptible to oxidative degradation during high-heat cooking.

## 4. Role of Meat Lipids in Human Health

### 4.1. Current Dietary Guidelines for Lipids

Current dietary guidelines from leading health organizations provide recommendations on total fat, saturated fats, *trans* fats, PUFAs and cholesterol to optimize health and prevent chronic diseases. These guidelines are informed by global research and are periodically updated to reflect emerging scientific evidence.

The World Health Organization (WHO) recommends that total fat intake should not exceed 30% of daily caloric intake for adults [26]. This range supports essential physiological functions, including energy provision and the absorption of fat-soluble vitamins, while minimizing risks associated with overconsumption [27]. Similarly, the Dietary Guidelines for Americans (DGA) align with this recommendation, promoting balanced fat consumption as part of healthy dietary patterns [28].

For saturated fats, the American Heart Association (AHA) and the WHO advise limiting intake to less than 10% of total daily calories to reduce CVD risk [29]. The AHA suggests a stricter target of less than 7% for individuals with elevated CVD risk [30]. These recommendations are based on evidence showing that replacing saturated fats with unsaturated fats, especially polyunsaturated fats, improves lipid profiles and reduces CVD risk [31]. The European Society of Cardiology (ESC) reinforces this guideline, emphasizing the importance of dietary patterns rich in plant-based foods and healthy fats [32].

The WHO and the Food and Agriculture Organization (FAO) have been leading the fight to eliminate industrially produced *trans* fats, recommending that *trans* fats intake be less than 1% of total daily calories [29,33]. This aligns with the AHA’s goal of completely removing artificial *trans* fats from the food supply, as these fats are strongly linked to elevated low-density lipoprotein (LDL) cholesterol and heightened CVD risk [34].

The balance of omega-6 to omega-3 fatty acids (*n*-6/*n*-3 ratio) is another focal point. Dietary recommendations advocate for an *n*-6/*n*-3 ratio of 4:1 or lower to maintain inflammatory balance and metabolic health [35,36]. However, modern diets often have ratios as high as 15:1, exacerbating inflammation and increasing the risk of chronic diseases like CVD and diabetes [37].

Regarding cholesterol, historical recommendations from the AHA and the DGA advised limiting dietary cholesterol to less than 300 mg per day [28,30]. However, recent guidelines, including the 2020–2025 DGA, have removed a specific limit, focusing instead on promoting dietary patterns naturally low in cholesterol [38]. This shift reflects evidence that dietary cholesterol has minimal impact on blood cholesterol levels for most individuals, although excessive intake alongside saturated fats may increase CVD risk in sensitive populations.

Dietary guidelines also emphasize minimizing exposure to lipid oxidation products due to their potential links to oxidative stress, inflammation and chronic diseases like CVD and cancer. Recommendations include reducing the consumption of fried and processed foods, which are significant sources of oxidized lipids, and adopting low-temperature cooking methods such as steaming or sous vide to limit oxidation during preparation. Protein lipoxidation, a non-enzymatic *post*-translational modification, occurs when reactive lipid oxidation products, such as malondialdehyde (MDA) and 4-hydroxy-2-nonenal (HNE), interact with amino acid residues in proteins, leading to the formation of adducts and cross-links [39]. This process significantly affects meat quality by altering texture, reducing water-holding capacity, contributing to off-flavours, and decreasing the bioavailability of essential amino acids. Lipoxidation also exacerbates oxidative stress by creating a feedback loop between lipid and protein oxidation, particularly during storage and processing under oxidative conditions. Effective mitigation strategies, such as antioxidant use, advanced packaging, and optimal storage conditions, are critical to preserving the sensory and nutritional properties of meat products. Consuming antioxidant-rich foods like fruits, vegetables and herbs, as well as avoiding reheated oils, further mitigate the risks. Fresh and minimally processed meats are encouraged, while the food industry’s use of antioxidants like vitamin E and selenium in meat production aligns with these goals. Oxidized lipids, such as malondialdehyde, are implicated in atherosclerosis and other chronic conditions, highlighting the importance of dietary practices to reduce their intake [27,31]. Usually, 0.4–0.6 mg of vitamin E per gram of PUFA is required to effectively protect from oxidative damage in biological systems and food products [40,41].

Table 2 provides an overview of dietary recommendations for various lipid components, including total fat, saturated fats, *trans* fats, polyunsaturated fats, the *n*-6/*n*-3 PUFA ratio and cholesterol. The guidelines are based on recommendations from leading health organizations, and the health rationales and relevant references are included to provide context and support for each guideline.

### 4.2. Dietary Contribution of Meat Lipids

The nutritional contributions of meat lipids are extensive, providing essential fatty acids, bioactive compounds and energy, while also presenting risks when consumed in excess or unfavourable compositions. These lipids influence metabolic, inflammatory and cognitive pathways, making their role in human health both significant and complex. Understanding the balance between their benefits and risks is essential for informed dietary decisions.

Meat lipids are a vital source of essential fatty acids, including omega-3 PUFAs such as EPA and DHA. These fatty acids are indispensable for human health, supporting cardiovascular function, cognitive development and inflammatory regulation. Grass-fed ruminant meat offers an alternative omega-3 source for populations with limited fish consumption, providing up to 1.5 g of omega-3 fatty acids per 100 g of muscle, significantly higher than the less than 0.5 g found in grain-fed meat [3].

In addition to EPA and DHA, meat lipids supply linoleic acid and ALA, precursors to omega-6 and omega-3 fatty acids, respectively. These precursors are crucial for maintaining immune response and cellular integrity. Ensuring an adequate intake of these nutrients through diverse dietary sources, including high-quality meat, can prevent deficiencies and associated health risks.

Beyond essential fatty acids, meat lipids contain bioactive compounds that contribute unique health benefits. CLA, predominantly found in ruminant meat, has been linked to improved lipid profiles, enhanced body composition and potential anti-carcinogenic properties [8]. CLA influences metabolic health by modulating lipid and glucose metabolism, with grass-fed animals producing higher CLA concentrations (0.3–0.8 g per 100 g) compared to grain-fed animals [42].

Phospholipids, another key component of meat lipids, play an integral role in maintaining cell membrane integrity, fluidity and signal transduction. These lipids are rich in PUFAs, particularly DHA, which supports brain function and cognitive health. Recent studies highlight the potential of phospholipids to reduce the risk of neurodegenerative diseases, including Alzheimer’s, through anti-inflammatory and neuroprotective pathways [43]. Branched-chain fatty acids, though present in smaller quantities, exhibit immunomodulatory properties and may aid in metabolic regulation, further expanding the functional benefits of meat lipids [44].

Despite their benefits, meat lipids also pose risks when consumed in excess or unfavourable compositions. SFAs, which constitute 40–50% of meat’s fatty acid profile, have historically been associated with increased LDL-cholesterol levels and a higher risk of CVD. However, recent meta-analyses suggest that the overall dietary context, including macronutrient substitutions (e.g., replacing SFAs with unsaturated fats instead of carbohydrates), plays a more critical role in determining health outcomes [9].

Dietary cholesterol, which constitutes 0.3–0.5% of total meat lipids, has also been linked to dyslipidaemia and atherosclerosis in sensitive individuals. However, its impact varies based on genetic predisposition and baseline dietary intake. Modern guidelines from organizations like the AHA advocate for moderation rather than strict avoidance of cholesterol, emphasizing its limited impact on blood lipid levels for most individuals [45].

Modern diets often feature an *n*-6/*n*-3 ratio exceeding 15:1, far above the recommended 4:1 ratio for inflammatory balance and optimal metabolic health [35]. This imbalance can exacerbate chronic inflammation and increase the risk of diseases like diabetes and CVD. Grass-fed meat, with a more favourable *n*-6/*n*-3 ratio, offers a practical dietary strategy to restore balance and mitigate these risks [37]. Incorporating omega-3-enriched meat products and diversifying dietary sources of omega-3s can further support this balance.

### 4.3. Influence of Cooking Methods on Meat Lipids

Cooking methods significantly impact the nutritional quality and health implications of meat lipids by inducing lipid oxidation and the formation of toxic compounds. High-temperature methods such as grilling, frying, and roasting accelerate lipid oxidation, leading to the generation of reactive compounds like MDA and HNE. These compounds are known for their pro-inflammatory and cytotoxic properties, contributing to oxidative stress and increasing the risk of chronic diseases such as cardiovascular disease and cancer [46].

To minimize these effects, alternative cooking techniques like sous vide and steaming are recommended. Sous vide cooking, which involves low temperatures over an extended period, preserves the nutritional quality of meat lipids and reduces oxidation. Additionally, using antioxidant-rich marinades containing herbs like rosemary or spices can effectively decrease the formation of oxidized lipids during cooking.

The consumption of oxidized lipids has been associated with adverse health outcomes due to their potential to disrupt cellular functions and promote atherosclerosis. Incorporating low-temperature cooking methods and antioxidant strategies not only enhances the safety and nutritional profile of meat but also aligns with dietary recommendations to reduce exposure to harmful compounds.

To summarize the diverse roles of meat lipids and their implications for human health, Table 3 provides an overview of key lipid components found in meat, their nutritional contributions and associated health implications. This table highlights the dual nature of meat lipids, outlining both their essential benefits, such as providing omega-3 fatty acids and phospholipids and potential risks linked to saturated fats and lipid oxidation products. Practical recommendations are also included to guide consumers and policymakers in optimizing meat lipid consumption for improved health outcomes.

## 5. Recent Advances and Innovations

Ongoing research and technological advancements have significantly expanded our understanding of meat lipids, paving the way for innovations that optimize their nutritional profiles. Recent developments focus on modifying the lipid content through animal feeding strategies, producing functional meat products, and improving analytical techniques to better characterize lipid profiles.

Dietary manipulation of livestock has proven to be one of the most effective strategies for improving the lipid composition of meat. Feeding animals omega-3-rich supplements, such as flaxseed, fish oil, microalgae or marine-based products, significantly enhances the levels of omega-3 PUFAs in meat [47,48,49,50]. For instance, omega-3-enriched diets in ruminants have been shown to triple the concentrations of EPA and DHA in muscle tissues compared to standard grain-based diets [3]. Similarly, chickens supplemented with flaxseed oil demonstrate a significant increase in ALA, a precursor of EPA and DHA, making poultry meat a viable source of omega-3 PUFAs for consumers [5].

Grass-based feeding systems inherently produce meat with a more favourable *n*-6/*n*-3 ratio, often below the recommended threshold of 4:1. In contrast, grain-fed systems can exhibit *n*-6/*n*-3 ratios as high as 15:1, which is associated with increased inflammation and chronic disease risk [37]. Furthermore, the incorporation of antioxidants like vitamin E and selenium in animal diets has been shown to mitigate lipid oxidation, preserving the integrity of PUFAs during meat storage and processing [51]. These antioxidants also improve meat shelf life and reduce the formation of harmful lipid peroxides during high-temperature cooking [13].

Emerging research is also exploring novel feed additives, such as bioactive compounds derived from seaweed or fermented by-products, which can further enhance the lipid quality of meat while promoting sustainability in livestock production [52,53,54]. Functional meat products aim to deliver health benefits beyond basic nutrition by incorporating bioactive ingredients or modifying lipid compositions. Structured lipids, such as emulsion gels and oil-bulking agents, are increasingly being used to create healthier meat products with reduced SFAs and enhanced MUFAs and PUFAs [55].

Post-harvest lipid modification strategies are gaining popularity. For example, incorporating omega-3-rich oils, such as flaxseed or algae oil, into marinades has successfully enriched the PUFA content of meat without altering its sensory qualities. These omega-3-enriched meat products offer consumers a practical way to meet dietary recommendations for essential fatty acids while maintaining the flavour and texture of traditional meat products [3].

Advancements in meat processing technologies are significantly enhancing the nutritional quality and safety of meat lipids by addressing key challenges such as lipid oxidation, microbial contamination, and nutritional degradation. High-pressure processing (HPP) inactivates microorganisms while retaining the nutritional integrity of lipids, making it a reliable method for maintaining meat quality during storage [56]. Similarly, pulsed electric fields (PEF) enhance microbial safety and stabilize lipids by minimizing the formation of harmful oxidation products, which can degrade nutritional value and flavour [57].

Encapsulation techniques, such as the use of biopolymers or liposomes, play a vital role in protecting unsaturated fatty acids from oxidative damage [58]. These methods not only extend the shelf life of meat products but also preserve the bioavailability of critical nutrients like omega-3 fatty acids during storage and cooking. Lipidomics, combined with advanced analytical tools like Raman and infrared spectroscopy, enables precise, real-time monitoring of lipid profiles throughout the processing chain. These technologies provide deeper insights into lipid oxidation pathways and allow for immediate adjustments to ensure product quality. Lab-grown meat, produced through cell culture techniques, has emerged as a future food source addressing sustainability, nutrition, and ethics [59]. It offers customizable lipid profiles, improved omega-6 to omega-3 ratios, reduced saturated fats, and the inclusion of bioactive compounds like conjugated linoleic acid (CLA). This innovation reduces environmental impacts, such as greenhouse gas emissions and water use, while eliminating animal welfare concerns. Despite challenges like high costs, scalability, and regulatory hurdles, lab-grown meat holds transformative potential for the food industry, warranting further research and development.

The development of hybrid meat products, which combine animal-derived meat with plant-based ingredients, is another innovative approach. These products often incorporate plant oils high in PUFAs, such as olive or canola oil, to optimize the lipid profile while reducing the overall SFA content. For example, burger patties made with a blend of beef and soybean oil demonstrate a healthier fatty acid composition without compromising taste or texture. Additionally, fortified meat products containing CLA, plant sterols or polyphenols are being developed to target specific health outcomes, such as reducing cholesterol levels or combating inflammation. These functional products align with consumer demands for food products that actively contribute to health and wellness [60].

The field of lipidomics has undergone substantial advancements, enabling more detailed characterization of meat lipids. Modern techniques, including gas chromatography (GC), high-performance liquid chromatography (HPLC) and mass spectrometry (MS), allow for precise quantification of individual fatty acids, such as omega-3 and CLA [61]. These methods provide critical insights into the nutritional quality of meat and its lipid-derived bioactive compounds. Recent innovations in the classical vibrational spectroscopy, such as Raman and infrared (IR) spectroscopy, have improved the analysis of lipids in situ, offering a rapid and non-destructive assessment of lipid composition. These tools are particularly valuable for monitoring lipid oxidation during meat storage and processing [62]. Data analytics and machine learning are also being integrated into lipidomic studies to predict the behaviour of lipids under various processing and storage conditions. These advancements enable more accurate modelling of lipid oxidation pathways, helping producers develop strategies to maintain the nutritional and sensory quality of meat.

## 6. Controversies and Research Gaps

Despite significant advancements in understanding meat lipids and their role in human health, several controversies and knowledge gaps remain. These issues revolve around conflicting evidence regarding saturated fats, population-specific dietary effects and the impact of processing methods. Addressing these gaps is essential for refining dietary guidelines and improving public health outcomes.

The role of SFAs in CVD has been a subject of ongoing debate. SFAs have traditionally been associated with elevated low-density lipoprotein (LDL) cholesterol levels and increased CVD risk, forming the basis for dietary guidelines limiting saturated fat intake to less than 10% of total energy [63]. However, recent meta-analyses and systematic reviews have provided a more nuanced perspective. Some studies indicate that the effects of SFAs on CVD are influenced by the macronutrients used as replacements. For instance, replacing SFAs with PUFAs consistently improves lipid profiles and reduces cardiovascular events, while substituting SFAs with refined carbohydrates offers no benefit [64,65].

Additionally, variations in individual responses to dietary saturated fats, particularly the type of SFA, further complicate this relationship. Stearic acid, abundant in beef, is considered neutral in its effects on serum cholesterol, contrasting with palmitic acid, which has more pronounced atherogenic effects [66]. This underscores the importance of dietary patterns and macronutrient interactions rather than focusing solely on saturated fat intake [10].

Dietary lipids have differential impacts across populations due to genetic variations, baseline nutrient intake and dietary patterns. For example, omega-3 PUFAs, such as EPA and DHA, are beneficial for cardiovascular and neurological health, but their effects may vary depending on the population’s baseline omega-3 intake and genetic predispositions [63]. Populations with higher fish consumption, such as those in coastal regions, exhibit lower CVD risks compared to populations reliant on grain-fed meats with less favourable *n*-6/*n*-3 ratios [3].

Epidemiological studies on meat lipids and chronic diseases, including diabetes and cancer, often yield inconsistent results due to variations in study design, confounders and dietary assessment methods. Expanding research to diverse populations and non-Western dietary contexts is critical for developing globally relevant dietary recommendations [63].

The way meat is cooked and processed significantly affects its lipid composition and health implications. High-temperature cooking methods, such as grilling and frying, accelerate lipid oxidation, producing harmful compounds like malondialdehyde, which are associated with oxidative stress and chronic diseases [65]. While sous vide cooking and antioxidant-rich marinades have shown potential in mitigating lipid oxidation, the long-term effects of oxidized lipids on health remain inadequately studied [13]. Additionally, the interaction of oxidized lipids with other meat components, such as heme iron and proteins, may exacerbate oxidative stress, further emphasizing the need for innovative cooking techniques and preservation methods to improve meat safety and nutritional value [10].

Addressing these controversies requires robust, high-quality studies that evaluate the complex interactions between dietary lipids, genetic predispositions and health outcomes. Research should prioritize the role of hybrid and plant-based meat products in diversifying dietary lipid sources, as well as innovations in lipidomics for precise profiling of lipid structures and their biological roles. Moreover, there is a pressing need for population-specific studies to develop tailored dietary recommendations, particularly for regions with unique dietary practices and nutritional needs [67].

## 7. Conclusions and Future Perspectives

Meat lipids are essential contributors to human health, offering vital nutrients such as essential fatty acids, bioactive compounds and energy. Their roles in metabolic regulation, inflammatory balance and cognitive health underscore their importance in the diet. However, their dual nature, as both beneficial nutrients and potential risk factors, requires careful consideration. Advances in animal feeding strategies, hybrid meat products and lipidomics have enhanced the quality of meat lipids and deepened our understanding of their health impacts. Despite these advancements, controversies around saturated fats, dietary cholesterol and lipid oxidation remain unresolved.

Consumers are encouraged to prioritize grass-fed or omega-3-enriched meat to achieve healthier fatty acid profiles, particularly improved *n*-6/*n*-3 ratios. Opting for leaner cuts of meat and reducing the intake of processed meat products can further minimize health risks. Additionally, low-temperature cooking methods, such as sous vide or steaming, are recommended to reduce lipid oxidation and preserve nutritional quality. Public health initiatives should focus on promoting dietary balance, particularly emphasizing the consumption of PUFAs and the importance of maintaining a low *n*-6/*n*-3 ratio. Policies supporting sustainable livestock practices, including grass-fed systems and the use of algae-based feeds, can enhance meat quality while reducing the environmental impact of meat production.

Future research should address controversies surrounding saturated fats, exploring their specific roles in cardiovascular and metabolic health, while population-specific studies can inform tailored dietary recommendations based on genetic and dietary variability. Innovation in functional meat products enriched with bioactive lipids like omega-3s, CLA and phospholipids, as well as hybrid meat products combining plant-based ingredients, offers opportunities to improve health outcomes while maintaining sensory qualities. Advancements in lipidomics and analytical techniques will enhance the monitoring of lipid profiles and oxidative changes, ensuring nutritional quality and safety. Sustainability-focused efforts, such as integrating algae and seaweed into livestock feed, promise to improve meat quality while reducing environmental impact, aligning with public health and ecological goals.

## Figures and Tables

**Table 1:** Description of lipid composition and differences across animal species.

Species	Triglycerides (%)	Phospholipids (%)	Cholesterol (mg/100 g)	Saturated Fats (%)	Monounsaturated Fats (%)	Polyunsaturated Fats (%)	Omega-6:Omega-3 Ratio	Bioactive Lipids
Fish [18]	5–10	15–20	30–50	20–30	25–40	30–50	1:1 to 3:1	High in omega-3 PUFAs
Cattle [6]	85–90	5–10	60–80	40–50	40–50	5–10	4:1 to 8:1	Moderate CLA
Sheep [8]	80–85	10–15	60–80	45–55	35–45	5–10	4:1 to 7:1	High CLA
Pigs [14]	90–95	5–8	50–70	35–45	40–50	10–15	8:1 to 12:1	Low CLA
Chicken [5]	90–95	5–10	50–70	30–40	45–50	10–20	10:1 to 15:1	Low bioactive lipids

**Table 2:** Summary of dietary guidelines for lipids in human diet.

Component	Guideline	Health Rationale	References
Total Fat	20–35% of daily energy intake for adults	Supports essential functions, including energy provision and absorption of fat-soluble vitamins	[28,29]
Saturated Fatty Acids (SFAs)	<10% of daily energy intake (AHA recommends <7% for high-risk populations)	Reduces cardiovascular risk by lowering LDL cholesterol	[30,32]
*Trans* Fatty Acids	<1% of daily energy intake; eliminate industrially produced *trans* fats	Strongly linked to increased LDL cholesterol and cardiovascular risk	[29,33]
Polyunsaturated Fatty Acids (PUFAs)	6–11% of total energy intake (WHO)	Essential for inflammatory and metabolic balance; supports cardiovascular and cognitive health.	[27,29]
Omega-6 to Omega-3 (*n*-6/*n*-3) Ratio	≤4:1 recommended for maintaining inflammatory balance and metabolic health	High ratios (>10:1) are associated with increased chronic inflammation and metabolic disorders	[35,36]
Cholesterol	Moderation; no specific limit in recent guidelines	Limited impact on serum cholesterol for most individuals; excessive intake in sensitive populations may increase CVD risk	[28,30]
Lipid Oxidation Products	Minimize exposure by reducing consumption of fried and processed foods; adopt low-temperature cooking methods	Oxidized lipids are linked to oxidative stress, inflammation and chronic diseases such as atherosclerosis and cancer	[27,31]

**Table 3:** Nutritional contributions and possible health implications of meat lipids.

Lipid Component	Sources in Meat	Nutritional Role	Potential Health Implications	Recommendations
Triglycerides	Intramuscular and subcutaneous fat	Primary energy source (9 kcal/g); carrier for fat-soluble vitamins	Excessive intake is linked to obesity and cardiovascular disease (CVD) risks [3]	Balance intake with lean cuts and plant-based fats
Phospholipids	Muscle cell membranes	Support cell membrane fluidity; involved in cognitive and neural functions	Potential benefits for neurodegenerative disease prevention [21]	Prioritize minimally processed meat with natural phospholipids
Cholesterol	All meat types	Precursor for hormones, bile acids, and vitamin D synthesis	Minimal impact on serum cholesterol in most individuals [38]	Moderate intake, prioritize natural sources
Saturated Fatty Acids (SFAs)	Fatty cuts, processed meat	Energy provision; contributes to lipid profiles	Excessive intake is linked to elevated LDL cholesterol in sensitive individuals [10]	Limit intake to <10% of total energy as per dietary guidelines.
Omega-3 PUFAs (EPA, DHA)	Grass-fed meat, enriched meat	Anti-inflammatory; supports cardiovascular and cognitive health	Low intake increases risk of inflammation, CVD, and neurodegenerative diseases [5]	Include omega-3 enriched or grass-fed meat in diets
Omega-6 PUFAs	Grain-fed meat	Essential for immune response and cellular integrity	High *n*-6/*n*-3 ratio linked to chronic inflammation [37]	Aim for a dietary *n*-6/*n*-3 ratio ≤ 4:1
Conjugated Linoleic Acid (CLA)	Ruminant meat	Potential anti-carcinogenic and lipid-lowering effects	Inconsistent evidence on therapeutic efficacy [8]	Prioritize ruminant meat from grass-fed animals
Lipid Oxidation Products	High-heat processed meat	Pro-inflammatory; linked to oxidative stress and chronic diseases	Long-term impacts under-studied [13]	Use low-temperature cooking methods (e.g., sous vide)

## Data Availability

No new data were created or analysed in this study. Data sharing is not applicable to this article.
